# Multidisciplinary management of intracardiac tumor thrombus in low-grade endometrial stromal sarcoma: case report and literature review

**DOI:** 10.3389/fonc.2025.1671080

**Published:** 2025-10-22

**Authors:** Hanke Zhang, Jinhua Chen, Chao Yang, Si Chen, Miao Wang, Xiaoyan Xin, Hongbo Wang, Xiaowu Zhu

**Affiliations:** ^1^ Department of Obstetrics and Gynecology, Union Hospital, Tongji Medical College, Huazhong University of Science and Technology, Wuhan, China; ^2^ Department of Vascular Surgery, Union Hospital, Tongji Medical College, Huazhong University of Science and Technology, Wuhan, China; ^3^ Department of Cardiovascular Surgery, Union Hospital, Tongji Medical College, Huazhong University of Science and Technology, Wuhan, China; ^4^ Department of Urology, Union Hospital, Tongji Medical College, Huazhong University of Science and Technology, Wuhan, China

**Keywords:** low-grade endometrial stromal sarcoma, metastasis, right atrium, cytoreductive surgery, multidisciplinary management

## Abstract

The low-grade endometrial stromal sarcoma (LG-ESS) represents a slowly proliferating subtype of endometrial stromal tumor but exhibits a preference for late-stage recurrence. Incidence of invasion into major blood vessels and the heart is exceedingly uncommon in this particular neoplasm. We describe a patient with recurrent LG-ESS whose tumor mainly involved the right ovary and right kidney and extended from the ovarian vein to the right atrium. Furthermore, we comprehensively review existing literature on 35 patients exhibiting similar manifestations. In these 36 cases, tumor thrombus primarily extended to the inferior vena cava (n=17), with a minority extending to the right atrium (n=12), while no cases were detected when the tumor thrombus had just extended to the iliac blood vessels, indicating that LG-ESS exhibits an insidious onset during early-stage vascular invasion. It is advisable for comprehensive examination and diligent follow-up in high-risk patients. 19 cases with complete tumor resection were followed up, and all except two patients remained alive with no evidence of disease. Among the 5 cases of mortality, 2 cases did not undergo surgical intervention, while in 3 cases where surgery was performed, complete tumor resection could not be achieved. The causes of death in these patients were related to disease progression or concurrent cardiovascular events. These findings underscore the importance of cytoreductive surgery adhering to the tumor-free principle, or at least removing the lesions in the major blood vessels and heart to prevent acute embolism and sudden demise, which usually requires multidisciplinary teamwork.

## Introduction

The endometrial stromal tumor (EST) is an uncommon subtype of stromal tumors characterized by intricate morphological and molecular heterogeneity (1, 2). According to the tumor classification of the World Health Organization (WHO) in 2020, EST can be categorized into four distinct groups: endometrial stromal nodules (ESN), low-grade endometrial stromal sarcoma (LG-ESS), high-grade endometrial stromal sarcoma (HG-ESS), and undifferentiated uterine sarcoma (UUS) (3). LG-ESS is the less aggressive subtype of EST and predominantly affects women in the perimenopausal stage. Patients with LG-ESS have a good prognosis and long-term survival but with a risk of late-stage recurrence (4, 5). LG-ESS typically consists of spindle-shaped cells resembling proliferative endometrial stromal cells, with tumor cells exhibiting swirling patterns surrounding spiral arteriole-like vessels. These cells typically exhibit minimal cytoplasmic content, while their nuclei are usually oval with limited mitotic activity (usually <5 per 10 high power fields; however, a higher count does not exclude this diagnosis) (6). Immunohistochemically, the majority of cases exhibited positive staining for estrogen receptor (ER), progesterone receptor (PR), and CD10. Molecular subtyping primarily involves the gene fusion of JAZF1-SUZ12 (formerly known as JJAZ1) (7, 8), followed by rearrangements involving PHF1 and multiple fusion partners such as JAZF1, EPC2, MBTD1, and MEAF6 (9–12). In light of the relatively low incidence of LG-ESS and the dearth of high-quality, large-scale clinical studies, a consensus regarding the optimal diagnostic and treatment strategy for LG-ESS is still lacking.

We describe a patient with recurrent LG-ESS whose tumor mainly involved the right ovary, right kidney, and extended from the ovarian vein to the right atrium. Furthermore, this study aims to provide a comprehensive review of existing literature on patients exhibiting similar manifestations, offering valuable insights for diagnosis and treatment strategies.

## Case report

**Figure 1:**
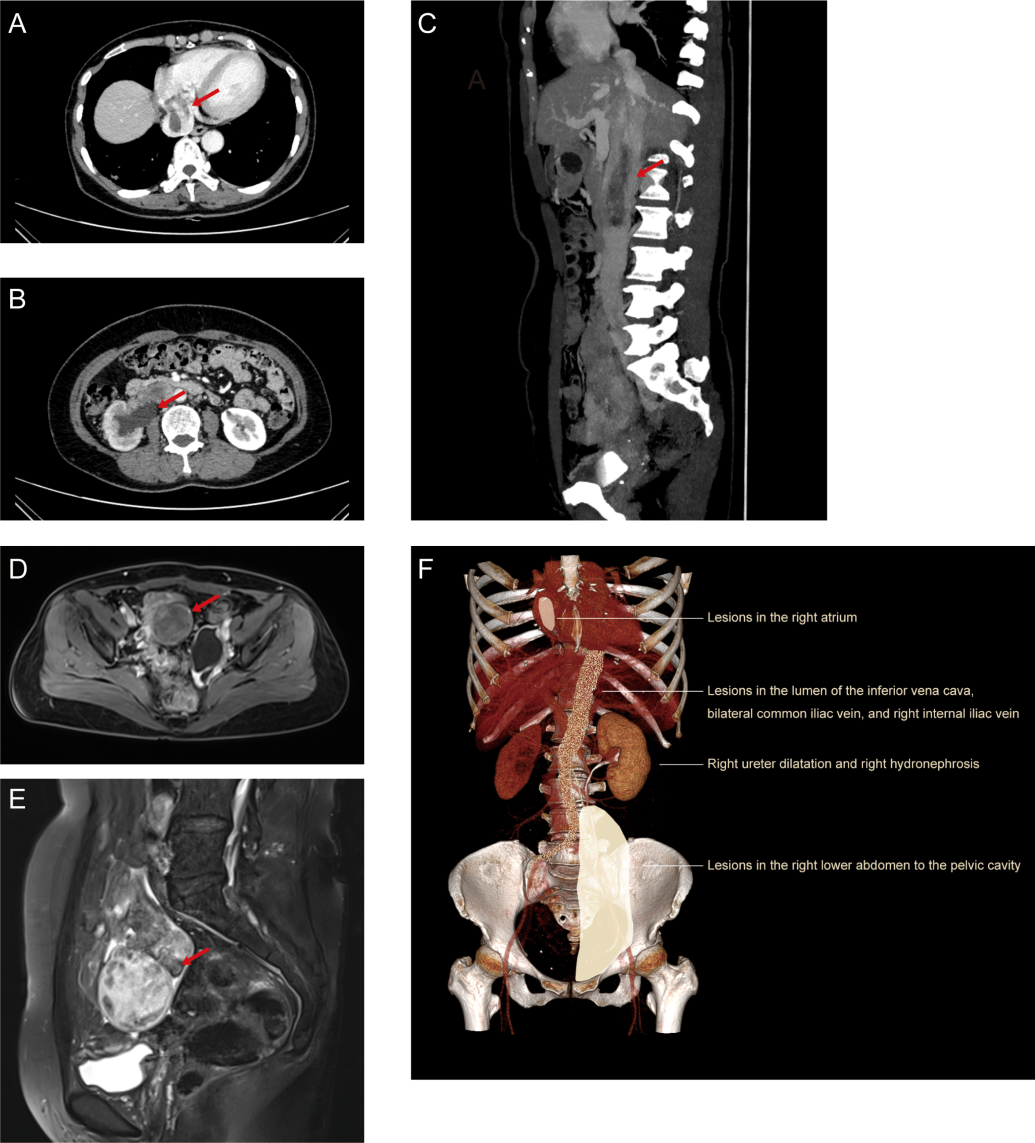
The imaging examinations of the tumor. **(A)** CTV showed the lesions in the right atrium. **(B)** CT showed dilation and hydrops in both the upper segment of the right ureter and right kidney. **(C)** CTV showed the lesions in the inferior vena cava. **(D, E)** MRI showed the lesions in the right lower abdomen to the pelvis. **(F)** The schematic depiction of three-dimensional imaging. The red arrow indicates the lesions.

We present a case of a 49-year-old female patient who underwent total abdominal hysterectomy for uterine fibroids at another medical facility 7 years prior. At the time of this visit, she reported symptoms of dizziness, chest tightness, and dull precordial pain, without palpitations, shortness of breath, or abdominal pain. The imaging examinations, including CT, MRI, and B-ultrasound, revealed a 7.5×5x11cm mass located in the right lower abdomen to the pelvis (**Figures 1D–F**). The demarcation with the lower segment of the right ureter (approximately at the level of the upper edge of the L4 vertebral body) appears indistinct. There is dilation and hydrops observed in both the upper segment of the right ureter and right kidney (**Figures 1B, F**). Strip-like filling defects are observed in the lumen of the right atrium, inferior vena cava, bilateral common iliac vein, and right internal iliac vein, along with localized luminal thickening of the inferior vena cava (**Figures 1A, C, F**). Although the definitive diagnosis of LG-ESS could not be made preoperatively, the imaging findings were highly suggestive of intravascular tumor thrombus. Specifically, CT and MRI demonstrated a large pelvic mass with associated hydronephrosis and continuous strip-like filling defects extending through the ovarian vein, inferior vena cava, and into the right atrium. These findings, combined with the patient’s prior hysterectomy and absence of other primary lesions, raised strong suspicion for recurrent LG-ESS. Differential diagnoses considered included intravenous leiomyomatosis (IVL), bland thrombus, and right renal malignancy with venous involvement. Among these, IVL was considered due to its known tendency to extend into pelvic veins and even the right atrium; however, IVL typically arises from uterine leiomyomas and lacks a dominant pelvic mass with invasive characteristics. Bland thrombus was less likely given the presence of a continuous enhancing soft-tissue mass extending from the ovarian vein to the right atrium, rather than a non-enhancing filling defect. Renal malignancy with venous invasion was also considered, but no primary renal mass was detected on imaging, and the tumor’s origin clearly traced from the right adnexal region. These imaging distinctions made LG-ESS the most likely diagnosis. Based on precise preoperative imaging and in consideration of the potential for abrupt cardiovascular events, the interdisciplinary team formulated a comprehensive surgical strategy prior to the operation with the aim of achieving complete resection of the pelvic mass and vascular lesions.

**Figure 2:**
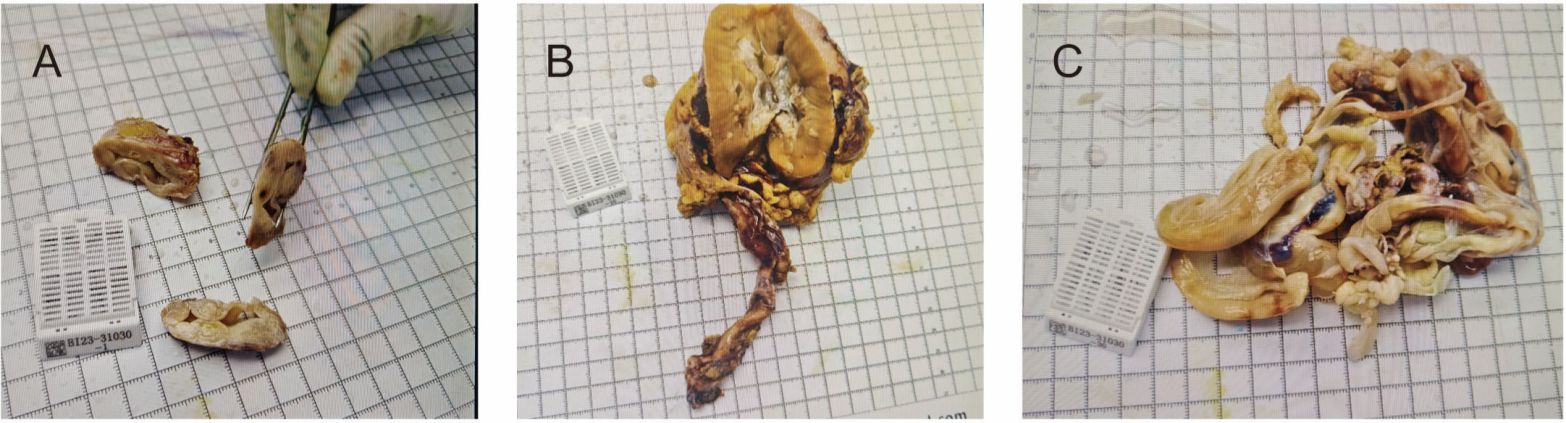
The resected specimens. **(A)** The left adnexa. **(B)** The right kidney specimen. **(C)** The intravascular tumor thrombus.

We performed a combined laparotomy and thoracotomy in collaboration with the cardiac, vascular, and urologic surgery teams. Intraoperatively, the right ovary was found to be markedly enlarged and elongated, measuring approximately 12 × 4 × 3cm, with a firm texture and prominent engorgement of surface vessels. The tumor extended from the ovarian vein within the infundibulopelvic ligament into the inferior vena cava (IVC), right renal vein, bilateral common iliac veins, right internal iliac vein, and further into the right atrium. The right ureter passed through the center of the tumor and was densely adherent, making separation impossible. We proceeded with bilateral salpingo-oophorectomy (BSO). The cardiac surgeon then performed a thoracotomy and pericardiotomy, initiated cardiopulmonary bypass, occluded the involved vein, incised the IVC, and successfully removed the tumor thrombus from both the IVC and the right atrium. Upon opening the atrium, no residual tumor was observed. However, during attempted extraction of the tumor thrombus from the right renal vein, it became evident that the lumen was completely occupied and the tumor was densely adherent to the venous wall, precluding complete resection. Following intraoperative consultation with the patient’s family, we collectively decided to proceed with right nephrectomy and partial ureterectomy to achieve complete tumor clearance. The vascular and urologic surgeons jointly performed the right nephrectomy, partial resection of the right ureter, and removal of tumor thrombus from the bilateral common iliac veins and right internal iliac vein. In summary, the surgical procedure included BSO, right nephrectomy with partial ureterectomy, pelvic tumor resection, and excision of tumor from the bilateral common iliac veins, IVC, right internal iliac vein, and right atrium. No visible residual tumor remained in the pelvis, abdomen, or thoracic cavity, achieving an R0 resection. Representative gross specimens are shown in **Figure 2**, including the left adnexa, the right kidney partially replaced by tumor, and the intravascular tumor thrombus. The right adnexa was submitted for intraoperative frozen section, and therefore no photograph was available.

**Figure 3:**
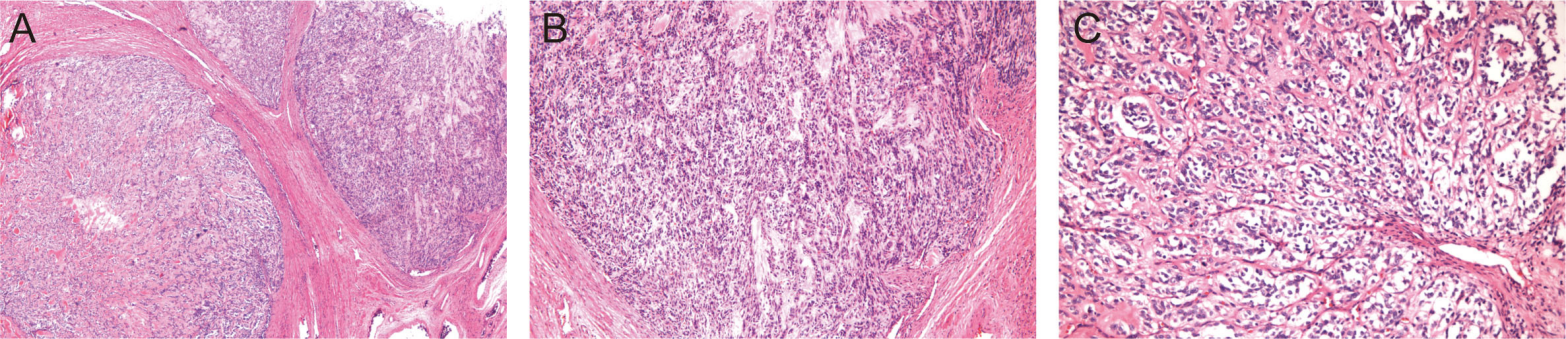
The HE staining of the tumor. **(A)** ×40. **(B)** ×100. **(C)** ×200.

The postoperative pathology report revealed that the tumor displayed a nodular growth pattern with variable nodule size, some with irregular margins. Within the nodules, tumor cells were arranged in nests and trabeculae, and areas of hyalinized stroma were observed. The tumor cells were small to medium in size, with hyperchromatic oval to spindle-shaped nuclei, inconspicuous nucleoli, uniform chromatin, mild atypia, and rare mitotic figures. These features were consistent with LG-ESS (**Figures 3A–C**). The right ovary was extensively replaced by the tumor. The tumor spread along the vessels accompanying the right ureter, involving the lumen of the right renal hilar area, and locally involved the right renal parenchyma along the vessels. Intravascular lesions exhibited tumor thrombus formation, accompanied by localized degenerative cystic changes. Immunohistochemical staining showed tumor cells: CD10(+), ER(+), PR(+), CyclinD1 (-), Desmin (partial +), H-caldesmon(-), P53 (individual +), PCK(-), PAX8(-), CD31 and CD34(indicating vascular tumor thrombus), Ki67(LI:10%). We contacted the hospital where the patient underwent the previous surgery, and upon thorough examination, the pathological diagnosis of the prior surgical procedure was rectified to be LG-ESS.

**Figure 4:**
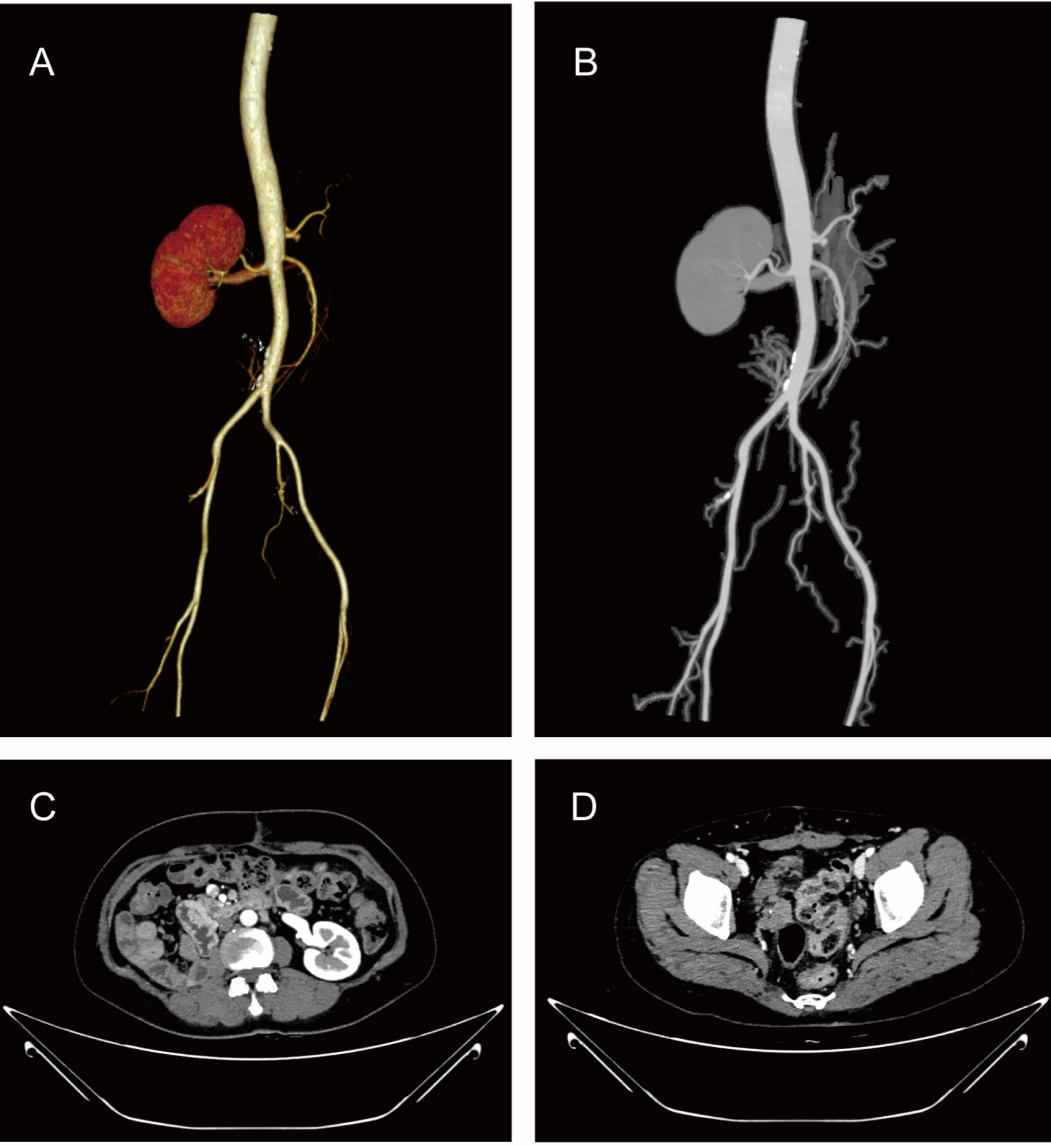
The imaging results at the five-month follow-up visit. **(A)** CTV three-dimensional reconstruction **(B)** CTV showing no obvious tumor thrombus in the inferior vena cava **(C)** Right nephrectomy specimen **(D)** No residual tumor tissue observed in the pelvic cavity.

The patient exhibited a satisfactory postoperative recovery. Although she was extensively counseled regarding adjuvant hormonal therapy, including its safety, tolerability, and evidence of recurrence reduction, but she firmly declined further treatment. Therefore, we adopted a close surveillance strategy with regular outpatient follow-ups. Comprehensive imaging was conducted at 5 months, including renal, vascular, and pelvic ultrasonography, cardiac echocardiography, and CT of the abdomen and pelvis with 3D reconstruction (**Figure 4**). A second follow-up was completed at 17 months postoperatively, including doppler ultrasonography of the hepato-biliary-pancreatic-splenic, renal and urinary tract, IVC and iliac veins, echocardiography, and transvaginal 3D ultrasound. Both follow-ups revealed no evidence of disease recurrence.

## Discussion

LG-ESS is a slowly growing neoplasia but has a propensity for late-stage recurrence. Invasion of main blood vessels and the heart is exceedingly rare in this tumor type. We identified 35 cases exhibiting similar tumor manifestations (**Table 1**), with an average age of 47.9±11.0 years (median: 48.5 years, range: 25–71 years) (13–37). Among the 18 patients with definite late recurrence, including this case, the average age was 48.0±9.8 years (median 49 years, range 30–63 years). Twelve of them were pathologically diagnosed as LG-ESS at the first visit and most of them underwent at least TH+BSO (n=9).

**Table 1:** 35 cases of LG-ESS with cardiovascular metastasis.

Reference	Primary/recurrent	Age	Previous treatment (primary pathological diagnosis) (corrected pathological diagnosis)	Maximal extension in the vessels	Other metastatic sites	Treatment	Follow-up time	Follow-up outcome (Cause of death)
(26)	Primary	55	-	PA	-	CRS (R0), HT	1y	ANED
(27)	Primary	70	-	IVC	Lymph nodes	Autopsy	-	Dead (acute pulmonary tumor thromboembolus)
(29)	Primary	48	-	RA	-	CRS (R2), chemotherapy	-	-
(33)	Primary	59	-	IVC	-	CRS (R0)	2m	ANED
(36)	Primary	58	-	RA	Parametrium, paracolpium	CRS (R2), HT	1y	ANED
(37)	Primary	56	-	IVC	Pelvic cavity	CRS (R0), radiotherapy	21m	ANED
(37)	Primary	28	-	IVC	-	CRS (R0), radiotherapy, HT	40m	AWD
(37)	Primary	43	-	RA	Pelvic cavity	CRS (R0)	20m	ANED
(37)	Primary	40	-	IVC	-	CRS (R0), radiotherapy, HT	13m	ANED
(37)	Primary	46	-	RV	-	CRS (R0), HT	6m	ANED
(14)	Recurrent	50	Uterine curettage, bladder biopsy, radiotherapy (ESS)	RA	-	CRS (R2), chemotherapy, radiotherapy	1y	Dead (progressive ascites, lower extremity, pulmonary edema)
(15)	Recurrent	52	TH (LG-ESS), recurrent tumor resection (LG-ESS), pulmonary wedge resection (LG-ESS)	RV	-	CRS (R0)	18m	ANED
(16)	Recurrent	63	TH, BSO, radiotherapy (ESS)	IVC	-	CRS	17m	ANED
(17)	Recurrent	49	TH, chemotherapy (uterine sarcoma) (LG-ESS), BSO, pelvic lesion resection, chemotherapy, radiotherapy (adult granulosa-cell tumor) (LG-ESS)	PA	-	CRS, chemotherapy, HT	3m	Alive
(18)	Recurrent	30	Myomectomy (hysteromyoma) (LG-ESS) TH, chemotherapy (LG-ESS) total pelvic exenteration, chemotherapy (LG-ESS)	IVC	On the surrounding of the external iliac artery	CRS	2y	AWD
(18)	Recurrent	43	TH (hysteromyoma) (LG-ESS)	LV	Vaginal vault, bladder, rectum, lung	Chemotherapy, radiotherapy	14y	Dead (heart failure)
(18)	Recurrent	49	TH (LG-ESS)	IVC	Bladder	Chemotheraoy, CRS (R0)	16m	ANED
(19)	Recurrent	45	TH, BSO, chemotherapy (LG-ESS)	IVC	-	CRS, chemotherapy, radiotherapy	6m	ANED
(21)	Recurrent	47	TH (hysteromyoma)	RA	-	CRS (R0), radiotherapy, HT	4.5y	ANED
(23)	Recurrent	59	TH, BSO, CRS, chemotherapy (LG-ESS)	AA	Retroperitoneum, lymph nodes	CRS, HT	6m	ANED
(24)	Recurrent	60	TH, BSO (LG-ESS)	IVC	-	CRS (R0)	1y	ANED
(25)	Recurrent	40	TH, BSO, omentectomy, IVC filter placement (LG-ESS)	RV	-	CRS (R0)	-	-
(28)	Recurrent	30	Unilateral salpingo-oophorectomy, chemotherapy (a low-grade malignant right ovarian tumor) (LG-ESS)	RA	-	CRS (thrombectomy), chemotherapy	-	-
(30)	Recurrent	49	TH, BSO, radiotherapy (LG-ESS)	RA	Right psoas, lung, lymph nodes	HT, chemotherapy	-	-
(31)	Recurrent	56	TH, BSO, hormonal treatment (LG-ESS)	IVC	Pelvic cavity	CRS (R0 not reached), chemotherapy	-	-
(34)	Recurrent	59	TH, BSO (LG-ESS)	RA	-	CRS (R0), HT	1y	Recurrent
(35)	Recurrent	34	Pelvic mass resection (LG-ESS)	IVC	Pelvic cavity	CRS (R0), HT	16m	ANED
(13)	Uncertain	25	TH, BSO (LG-ESS)	RA	Pelvic cavity	CRS (R0), radiotherapy	2y	ANED
(20)	Uncertain	38	Total pelvic exenteration (LG-ESS)	IVC	-	CRS (R2)	4y	Dead (multiple tumor metastasis)
(20)	Uncertain	48	TH (LG-ESS)	IVC	Bone, pelvic cavity	CRS (R2)	2y	Dead (multiple tumor metastasis)
(22)	Uncertain	71	TH, BSO	RA	-	CRS(R0)	-	-
(32)	Uncertain	38	Hysteroscopic submucosal myomectomy (hysteromyoma) (LG-ESS can’t be ruled out)	RA	-	CRS(R0), HT	3y	ANED
(37)	Uncertain	46	TH, BSO (hysteromyoma)	IVC	pelvic cavity	CRS(R0), HT	36m	ANED
(37)	Uncertain	51	TH (hysteromyoma)	IVC		CRS(R0), radiotherapy, HT	42m	ANED
(37)	Uncertain	41	TH, BSO (hysteromyoma)	IVC	pelvic cavity	CRS(R0)	98m	ANED

m, months; y, years; CRS, cytoreductive surgery; TH, total hysterectomy; BSO, bilateral salpingo-oophorectomy; HT: hormonal treatment; IVC, inferior vena cava; RA, right atrium; RV, right ventricle; PA, pulmonary artery; LV left ventricle; ANED, alive with no evidence of disease; AWD, alive with disease.

The columns are defined as follows:

Reference refers to the published literature source of the case.

Primary/Recurrent indicates whether the vascular involvement occurred at initial diagnosis (primary) or during disease relapse (recurrent).

Age represents the patient’s age at the time of diagnosis of vascular extension.

Previous treatment (Primary pathological diagnosis/Corrected pathological diagnosis) provides a brief summary of prior interventions (e.g., surgery), the initial pathological interpretation (which may have been incorrect), and the final corrected diagnosis upon review.

Maximal extension in the vessels denotes the most proximal anatomical site involved by tumor thrombus, such as the inferior vena cava or right atrium.

Other metastatic sites list any additional metastatic involvement outside the vascular system (e.g., lung, liver).

Treatment outlines the therapeutic approach, including surgical resection, hormonal therapy, chemotherapy, or radiotherapy.

Follow-up time is the duration of postoperative or post-treatment surveillance, typically reported in months or years.

Follow-up outcome (Cause of death) describes the clinical status at last follow-up, including disease recurrence, survival, or death with specified cause when available.

In these cases, tumor thrombus primarily extended to the inferior vena cava (n=17), with a minority extending to the right atrium (n=12) (including this case), and very few reaching the right ventricle (n=3), pulmonary artery (n=2), left ventricle (n=1), or abdominal aorta (n=1). Almost no cases were detected when the tumor thrombus had just extended to the iliac blood vessels, indicating that LG-ESS exhibits an insidious onset and lacks evident clinical symptoms during early-stage vascular invasion. A closely related entity, intravenous leiomyomatosis (IVL), may present with similar features—including tumor extension into the inferior vena cava and even the right atrium—and can pose similar diagnostic and surgical challenges. Although IVL is histologically benign, its intravascular growth pattern can mimic LG-ESS radiologically and clinically. Reports of IVL with intracardiac extension emphasize the importance of preoperative imaging and multidisciplinary planning in cases of suspected vascular tumor thrombi (38–40). Simultaneously, CT and MRI, as potent tools for early detection of cardiovascular involvement, may occasionally fail to accurately identify intravascular tumors (22, 32, 36), illustrating the significance of comprehensive preoperative assessment employing multiple imaging modalities and the consideration of periodic cardiovascular imaging during follow-up. In addition, targeted evaluation of the ovarian veins with contrast-enhanced CT may be particularly useful, as at least several reported cases, including ours, demonstrated tumor thrombus originating from the ovarian vein. Careful surveillance of this vascular segment during follow-up imaging may therefore facilitate earlier recognition of recurrence and guide timely surgical planning. Patients who may particularly benefit from such imaging examinations include those with a prior history of LG-ESS who underwent hysterectomy with ovarian preservation, individuals considered at high risk for recurrence, and patients presenting with unexplained venous or urinary tract abnormalities. Tailored imaging surveillance in these groups could enhance the likelihood of early detection and improve clinical outcomes. In light of the cases summarized in **Table 1**, several features may indicate a higher risk of recurrence. These include patients with ovarian preservation at the time of initial surgery, those who experience late recurrence beyond five years after hysterectomy, and individuals in whom tumor thrombus arises from the ovarian vein. At least 7 of these cases demonstrated a definite origin of tumor thrombus in the ovarian vein (13, 16, 18, 20, 31, 34) (including this case), highlighting the invasive potential of LG-ESS into blood vessels. Therefore, it may be advisable to consider complete resection of both ovarian veins or, at least, perform a high bilateral ligation (21). Recognizing these patterns provides valuable clinical implications for stratifying follow-up intensity and optimizing early detection strategies.

Surgery is the preferred treatment option for LG-ESS, irrespective of initial diagnosis or recurrence. Among the 32 cases that underwent cytoreductive surgery, at least 20 clearly demonstrated complete resection. Out of these cases with complete tumor resection, a follow-up was conducted for 19 cases with an average duration of 25.47±22.46 months (median 18 months, range 2–98 months). With the exception of two cases experiencing recurrence, all other patients remained alive with no evidence of disease. Among the 5 cases of mortality, 2 cases did not undergo surgical intervention, while in 3 cases where surgery was performed, complete tumor resection could not be achieved. The causes of death in these patients were related to disease progression or concurrent cardiovascular events. These findings underscore the importance of comprehensive tumor removal by a multidisciplinary surgical team for such patients, provided their physical condition permits. Despite its tendency for late-stage recurrence, surgical intervention can effectively mitigate the risk of severe complications such as heart failure, pulmonary embolism, and sudden death in patients with LG-ESS.

In addition, there remains ongoing debate regarding the management of LG-ESS, such as pertaining to the necessity of lymph node dissection. The incidence of lymph node metastasis in LG-ESS patients who underwent lymphadenectomy was found to be as low as 0-7% (41–44), suggesting that lymphatic invasion is not the primary route of metastasis in LG-ESS. Moreover, several studies have consistently demonstrated that lymph node metastasis and dissection don’t significantly impact overall survival rates in these patients (45–47), thereby discouraging routine systematic lymphadenectomy for the patients without evident lymph node enlargement. Adjuvant therapy, encompassing chemotherapy, radiotherapy, and hormonal therapy, represents another contentious element in the field. Multiple studies have demonstrated a lack of association between adjuvant chemotherapy and survival outcomes in patients with LG-ESS and it may even exert a detrimental effect on overall survival (42, 45, 48). Currently, there is insufficient evidence to support the use of adjuvant chemotherapy for LG-ESS, and no specific recommendation regarding the optimal chemotherapy regimen has been established. Also, limited clinical data exists regarding the efficacy of adjuvant radiotherapy. A retrospective study involving 152 patients with stage I to II LG-ESS demonstrated that the implementation of postoperative adjuvant radiotherapy resulted in a significant reduction in the incidence of pelvic recurrence, while concurrently improving the disease-free survival (49). However, radiotherapy may result in significant late toxicity, including the risk of secondary malignant tumors. Considering that LG-ESS is an indolent tumor, the efficacy and side effects of radiotherapy should be carefully evaluated. Moreover, some studies suggested that adjuvant radiotherapy does not provide any benefits for patients with LG-ESS (48, 50). The expression of estrogen and progesterone receptors is observed in approximately 70-80% of LG-ESS (51), leading to the proposal of various hormonal treatment options such as progestins, GnRHa, and aromatase inhibitors. Several small retrospective studies have demonstrated the potential benefits of adjuvant hormonal therapy in patients with LG-ESS, particularly in those advanced or relapsed patients (52, 53). In contrast, a large retrospective cohort analysis of 2414 patients with LG-ESS showed no overall survival benefit from adjuvant hormonal therapy, but only 12.7% of patients received hormonal therapy (n=307) (48). A meta-analysis of 10 retrospective studies demonstrated that adjuvant hormonal therapy significantly reduced the risk of recurrence in patients with LG-ESS while exhibiting limited impact on overall survival (54). In this case, subsequent to a comprehensive discussion regarding the advantages and disadvantages of diverse adjuvant therapies with the patient, her preference was forgoing any adjuvant treatment; therefore, we advised close follow-up of the patient.

This study has several limitations. Firstly, the molecular typing characteristics of this patient, such as the presence of genetic mutations, remain unknown and require further *in vitro* experimentation. Secondly, LG-ESS exhibits a propensity for delayed recurrence, however, we have conducted only one follow-up assessment thus far. Consequently, continuous follow-up of the patient’s survival status will be conducted.

## Conclusion

The occurrence of cardiac or large vessel metastasis in LG-ESS is a rare phenomenon, characterized by an insidious onset that poses challenges for early detection. Therefore, it is advisable to emphasize the importance of comprehensive examination and diligent follow-up in high-risk patients. The primary treatment option entails cytoreductive surgery adhering to the tumor-free principle, or at least removing the lesions in the major blood vessels and heart to prevent acute embolism and sudden demise. This approach typically necessitates collaborative efforts from a multidisciplinary team.
